# Novel Treatments of Adult T Cell Leukemia Lymphoma

**DOI:** 10.3389/fmicb.2020.01062

**Published:** 2020-05-28

**Authors:** Hiba El Hajj, Kunihiro Tsukasaki, Morgane Cheminant, Ali Bazarbachi, Toshiki Watanabe, Olivier Hermine

**Affiliations:** ^1^Department of Experimental Pathology, Microbiology, and Immunology, Faculty of Medicine, American University of Beirut, Beirut, Lebanon; ^2^Department of Hematology, International Medical Center, Saitama Medical University, Saitama, Japan; ^3^INSERM UMR 1163 and CNRS URL 8254, Imagine Institute, Paris, France; ^4^Department of Hematology, Necker-Enfants Malades University Hospital, Assistance Publique Hôpitaux de Paris, Paris-Descartes University, Paris, France; ^5^Department of Internal Medicine, Faculty of Medicine, American University of Beirut, Beirut, Lebanon; ^6^Department of Anatomy, Cell Biology and Physiological Sciences, Faculty of Medicine, American University of Beirut, Beirut, Lebanon; ^7^Department of Medical Genome Sciences, The University of Tokyo, Tokyo, Japan

**Keywords:** ATL, HTLV-1, arsenic, monoclonal antibodies, targeted therapies, epigenetic therapies, vaccine

## Abstract

Adult T cell leukemia-lymphoma (ATL) is an aggressive malignancy secondary to chronic infection with the human T cell leukemia virus type I (HTLV-I) retrovirus. ATL carries a dismal prognosis. ATL classifies into four subtypes (acute, lymphoma, chronic, and smoldering) which display different clinical features, prognosis and response to therapy, hence requiring different clinical management. Smoldering and chronic subtypes respond well to antiretroviral therapy using the combination of zidovudine (AZT) and interferon-alpha (IFN) with a significant prolongation of survival. Conversely, the watch and wait strategy or chemotherapy for these indolent subtypes allies with a poor long-term outcome. Acute ATL is associated with chemo-resistance and dismal prognosis. Lymphoma subtypes respond better to intensive chemotherapy but survival remains poor. Allogeneic hematopoietic stem cell transplantation (HSCT) results in long-term survival in roughly one third of transplanted patients but only a small percentage of patients can make it to transplant. Overall, current treatments of aggressive ATL are not satisfactory. Prognosis of refractory or relapsed patients is dismal with some encouraging results when using lenalidomide or mogamulizumab. To overcome resistance and prevent relapse, preclinical or pilot clinical studies using targeted therapies such as arsenic/IFN, monoclonal antibodies, epigenetic therapies are promising but warrant further clinical investigation. Anti-ATL vaccines including Tax peptide-pulsed dendritic cells, induced Tax-specific CTL responses in ATL patients. Finally, based on the progress in understanding the pathophysiology of ATL, and the risk-adapted treatment approaches to different ATL subtypes, treatment strategies of ATL should take into account the host immune responses and the host microenvironment including HTLV-1 infected non-malignant cells. Herein, we will provide a summary of novel treatments of ATL *in vitro*, *in vivo*, and in early clinical trials.

## Introduction

### Adult T Cell Leukemia: Epidemiology, Pathophysiology, Clinical Presentation and Classification

Adult T cell leukemia (ATL) was discovered in Japan in 1976 (Takatsuki et al., 1976), and was associated with the Human T cell leukemia virus type I (HTLV-I) in 1980 (Poiesz et al., 1981). ATL is an aggressive malignancy characterized by the clonal expansion of mature activated T cells generally CD3^+^ CD4^+^ CD5^+^ CD7^–^ CD8^–^ CD25^+^ (Uchiyama et al., 1983; Waldmann et al., 1984; Dasanu, 2011; Tian et al., 2011). It occurs in 1–5% of the infected individuals, after a very long latency period which may exceed 50 years (Gessain and Mahieux, 2000; Gessain, 2011; Ishitsuka and Tamura, 2014; Watanabe, 2017; Bangham, 2018). HTLV-I is the first oncogenic retrovirus allied with a human disease. It infects around 10–20 million people worldwide, and is endemic in several countries including Southern Japan, the Caribbean islands, Central and Latin America, Romania, the Middle East (chiefly Iran), Intertropical Africa, Melanasia, and Central Australia (Gessain and Mahieux, 2000; Laperche et al., 2009; Gessain, 2011; Bangham, 2018; Afonso et al., 2019). Alarmingly, a rising incidence of ATL was reported in non-endemic regions including the United States and the North of Japan (Chihara et al., 2012; Malpica et al., 2018).

The oligoclonal expansion of HTLV-I infected cells results from the expression of the viral transactivator Tax (Wattel et al., 1995; Mortreux et al., 2001). Tax oncoprotein plays a critical role in ATL initiation and leukemogenesis through alteration of many cellular pathways, following activation of the viral promoter, and creation of an autocrine loop involving interleukin-2 (IL-2), IL-15 and their respective receptors (Bamford et al., 1994; Wattel et al., 1995; Azimi et al., 1999; Mortreux et al., 2001). Tax activates CREB/ATF, AP-1, and NF-κB transcription factors, upregulates anti-apoptotic proteins, represses p53, DNA polymerase beta, PCNA, and MAD-1 checkpoints and interferes with several cell cycle regulators and DNA repair systems (Mulloy et al., 1998; Kfoury et al., 2005; Matsuoka and Jeang, 2011). Indeed, Tax binds to IKK-γ, the regulatory subunit of the IkappaB kinase (IKK) complex (Yamaoka et al., 1998; Harhaj and Sun, 1999; Kfoury et al., 2008; Wang et al., 2016), resulting in the activation of the NF-κB pathways and its downstream effector genes (Reviewed in Kfoury et al., 2005). This induced NF-κB activation highly depends on Tax post-translational modifications namely ubiquitylation, sumoylation, and urmylation (Nasr et al., 2006; Kfoury et al., 2008, 2011; Hleihel et al., 2018, Reviewed in Kfoury et al., 2012). Furthermore, Tax modulates the microenvironment and increases invasion and extravasation of ATL cells (El-Sabban et al., 2002; Bazarbachi et al., 2004a). Tax down-regulates the expression of various micro RNAs (mi-RNA) including mi-RNA31 known to inhibit the expression of the NF-κB non-canonical pathway components (Yamagishi and Watanabe, 2012). Tax transforms T cells *in vitro*, and its expression in T cells of transgenic mice, or in human CD34^+^ stem cells induces leukemia with striking ATL features recapitulating the human disease (Portis et al., 2001; Hasegawa et al., 2006; Banerjee et al., 2007; Ohsugi et al., 2007; Moodad et al., 2018). Tax also induced rough eye phenotype, a hallmark of malignant transformation in Drosophila models (Shirinian et al., 2015). Although Tax protein expression is very low in most ATL patients, recent *in vitro* data demonstrated that transient bursts of Tax expression occur sequentially in small fractions of ATL-derived cells (Billman et al., 2017). Importantly, ATL-derived cells depend on Tax expression for their long-term survival, even when Tax protein is undetectable by western blot (Dassouki et al., 2015; Mahgoub et al., 2018).

Another viral nuclear protein, HBZ, is encoded by the complementary strand of HTLV-I RNA genome (Larocca et al., 1989; Gaudray et al., 2002). HBZ is a negative regulator of Tax-mediated viral transcription (Gaudray et al., 2002), and its transcript levels positively correlate with HTLV-I proviral load in both ATL patients and asymptomatic carriers (Saito et al., 2009). Unlike Tax, HBZ is constantly expressed in ATL cells (Saito et al., 2009; Mahieux, 2015; Sugata et al., 2015). Although HBZ was shown to promote the proliferation of ATL cells *in vitro*, short hairpin RNA (shRNA) directed against HBZ only results in a modest inhibition of ATL cells proliferation, showing that HBZ is dispensable for the survival of ATL cells (Satou et al., 2006; Arnold et al., 2008). HBZ affects several cellular pathways implicated in cellular proliferation (Panfil et al., 2016). Unlike Tax, HBZ inhibits NF-κB, attenuating Tax effect and thus preventing ATL cells senescence (Panfil et al., 2016). One HBZ transgenic mouse model exhibited transforming features, yet without NF-κB activation, contrary to human ATL or Tax transgenics (Yoshida et al., 2008; Matsuoka and Green, 2009; Saito et al., 2009; Matsuoka and Jeang, 2011; Zhao and Matsuoka, 2012; Ma et al., 2016).

At the clinical level, ATL patients have a heterogeneous presentation from indolent slowly progressive disease to aggressive and life-threatening disease. The Shimoyama classification divides the disease into four major subtypes: smoldering, chronic, lymphoma, and acute (Shimoyama, 1991). All are characterized by a dismal long-term prognosis and a low median survival rate, ranging between 6 months for the acute subtype and 24 months for the chronic subtype (Shimoyama, 1991).

Acute ATL accounts for 55–60% of patients (Shimoyama, 1991; Pombo De Oliveira et al., 1999; Katsuya et al., 2015). It is characterized by major lymphocytosis of “flower cells” or “clover leaf” which are features typical of ATL, lymphadenopathy, hepatosplenomegaly, hypercalcemia, and its related symptoms such as renal dysfunction or neuropsychiatric disturbances (Tamura et al., 1986; Kiyokawa et al., 1987), high lactate dehydrogenase (LDH) (Yamaguchi et al., 1983) and frequent opportunistic infections due to a profound immunosuppression. These include pneumonia caused by *Pneumocystis carinii*, disseminated cryptosporidiosis and toxoplasmosis, cytomegalovirus, bacterial abscesses and sepsis as well as fungal infection and cytomegalovirus activation (Ueda et al., 1979; Blayney et al., 1983; Bunn et al., 1983; Verdonck et al., 2007). Skin and visceral lesions in the bone, the gastrointestinal tract and the lung may be also observed.

Lymphoma ATL accounts for around 20–25% of ATL cases (Shimoyama, 1991; Pombo De Oliveira et al., 1999; Katsuya et al., 2015). This form is characterized by the same symptoms of the acute subtype, but differs in presenting less than 1% leukemia cells in the peripheral blood, thus the absence of lymphocytosis. Smoldering ATL subtype accounts for 5–10% of cases (Shimoyama, 1991; Pombo De Oliveira et al., 1999; Katsuya et al., 2015) and is characterized by a normal leukocyte count but the presence of 1–5% flower cells. In addition, only skin and lungs may be infiltrated without the involvement of any other viscera. Finally, the chronic ATL subtype accounts for 10–20% of patients (Shimoyama, 1991; Pombo De Oliveira et al., 1999; Katsuya et al., 2015), and associates with a high leukocyte count causing lymphadenopathy and hepatosplenomegaly, absence of hypercalcemia, or visceral involvement, normal or slightly increased LDH levels. This subtype is further divided into favorable or unfavorable subgroups. The latter is defined by low serum concentration of albumin, high level of LDH, or high serum urea concentration, as well as high expression of Ki-67 antigen (Shirono et al., 1989; Yamada et al., 2001). It is worth noting that the term “indolent ATL” refers to smoldering and “favorable” chronic subtypes, while “aggressive ATL” includes the acute and lymphoma as well as the “unfavorable” chronic subtypes.

### Classical Therapeutic Options of ATL: Chemotherapy, Antiviral Therapy and Stem Cell Transplantation

Adult T cell leukemia remains a challenging malignancy. The clinical management of ATL and the proposed therapeutic options largely depend on the ATL subtype. The aggressive forms of ATL associate with a dismal prognosis, an intrinsic chemo-resistance, and a profound immunosuppression, associated with secondary opportunistic infections (Shimoyama, 1991; Hermine et al., 1998; Bazarbachi et al., 2004b, 2011; Tobinai, 2009). Patients with indolent ATL have a better prognosis. In Japan, these patients were managed with the watch-and-wait policy until progression or with chemotherapy, yet the long term results remain very poor (Takasaki et al., 2010; Hermine et al., 2018).

In addition to the Shimoyama classification, several prognostic factors were identified. For instance, elevated LDH levels, four total involved areas or more, thrombocytopenia, bone marrow involvement, eosinophilia, hypercalcemia, age of 40 or above, p53 mutations, p16 deletions, high IL-15 serum levels, and C-C chemokine receptor 4 (CCR4) expression are nowadays factors that predict poor prognosis (Tsukasaki et al., 2009). An international consensus report, published in 2009, defined prognosis factors, clinical features, therapeutic strategies, and response criteria for ATL (Tsukasaki et al., 2009). A recent update was recently published (Cook et al., 2019).

Standard treatment strategies of ATL include watch and wait policy for indolent ATL, conventional chemotherapy, the combination of two antiviral agents zidovudine (AZT) and interferon-alpha (IFN), and allogeneic hematopoietic stem cell transplantation (HSCT) (Tsukasaki et al., 2009; Bazarbachi et al., 2011; Hermine et al., 2018; Cook et al., 2019). The different Japanese trials using acute lymphoblastic leukemia or Non Hodgkin lymphoma -inspired chemotherapy regimen clearly demonstrated that ATL cells are chemo-resistant and that chemotherapy had little effect on the survival of ATL patients, specifically those with acute ATL (Dearden et al., 1991; Tanabe et al., 1991; Tobinai et al., 1992; Tsuda et al., 1994; Taguchi et al., 1996; Yamada et al., 2001; Tsukasaki et al., 2003; Takasaki et al., 2010). Although a phase 3 study in Japan demonstrated that the chemo-intensive LSG15 regimen was better than CHOP in newly diagnosed acute, lymphoma, or unfavorable chronic ATL, the CR rate was less than 20% for patients with acute ATL who received the LSG15 regimen and their 4 year survival was less than 10% (Tsukasaki et al., 2007). In the United States, a meta-analysis study including 195 ATL patients treated with modern therapies between 2000 and 2016, showed that the median survival rate was 4 months for acute ATL, 10 months for ATL lymphoma, 72 months for chronic/smoldering ATL, and not reached for unfavorable chronic type, with 4-year survival rates of 10, 4, 60, and 83%, respectively (Malpica et al., 2018). Overall, chemotherapy improved response rates in lymphoma but not in acute subtypes, yet with little impact on long-term survival.

Indolent smoldering or chronic ATL subtypes have a better prognosis than the aggressive ATL subtypes (Shimoyama, 1991). These variants were classically managed in Japan by a watchful-waiting policy (Takasaki et al., 2010), until progression of the disease or with chemotherapy when poor prognostic factors were identified (Tsukasaki et al., 2009). In a study of watch and wait for indolent ATL in Brazil over 14 years, the reported overall survival rates remained less than 20% at 5 years in both types (Bittencourt et al., 2007). Longer follow ups in Japan using this policy, showed that patients with smoldering ATL had an estimated 15-year survival rate of 13% with a median survival of 3 years, whereas patients with chronic ATL had an estimated 15-year survival rate of 15% with a median survival of 5 years (Takasaki et al., 2010). Thus, watch and wait strategy or treatment of the smoldering and chronic subtypes with chemotherapy still associate with a poor long-term outcome.

Important advances in the treatment of ATL were reported with the combination of two antiviral agents: AZT and IFN, which induced a high rate of response and became a standard treatment of ATL (Gill et al., 1995; Hermine et al., 1995; Bazarbachi and Hermine, 1996; Kchour et al., 2009; Bazarbachi et al., 2010, 2011; Malpica et al., 2018). The efficacy of this combination was confirmed, with minor differences, in various clinical trials and in a worldwide meta-analysis (Matutes et al., 2001; White et al., 2001; Hermine et al., 2002; Ratner et al., 2009; Bazarbachi et al., 2010). These results have transformed the clinical management of ATL in most parts of the world (Bazarbachi et al., 2010, 2011; Kinpara et al., 2013). Indeed, AZT/IFN proved highly effective and significantly improved survival in the leukemic chronic and smoldering subtypes of ATL as well as in a subset of the acute subtype with wild type P53 (Bazarbachi et al., 2010). Patients with the lymphoma subtype benefited from induction chemotherapy, when given simultaneously or sequentially with AZT and IFN (Hodson et al., 2011). Patients with previously untreated ATL or newly diagnosed ATL achieved higher response rates (Matutes et al., 2001; Hermine et al., 2002) compared to heavily treated patients (White et al., 2001). An ongoing randomized trial in Japan is testing AZT and IFN versus watchful waiting in patients with indolent ATL. Unfortunately, many patients are either resistant or progress even after a long period of disease control. Furthermore, treatment should be continued for life as relapse always occurs upon stopping therapy, indicating that AZT/IFN is not curative. In the United States, first-line AZT/IFN resulted in a median progression-free survival (PFS) in patients with aggressive ATL who achieved CR after AZT/IFN of 48 months versus 11 months after chemotherapy (Malpica et al., 2018), suggesting that AZT/IFN can be used against aggressive ATL, as up-front option followed by chemotherapy switch in non-responders. At the molecular level, this combination inhibited the reverse transcriptase activity and modified the clonality pattern in responding ATL patients (Macchi et al., 2017). Since reverse transcriptase mediated viral replication does not occur in the malignant cells, these results highly suggest that the primary target of the AZT/IFN combination is the ATL microenvironment, specifically *de novo* infection of T cells by HTLV-1 which appears critical for the survival of the malignant clone.

Because of the high rate of relapse after conventional chemotherapy, allogeneic stem cell transplantation (alloSCT) is an attractive potentially curative option (Iqbal et al., 2019). However, most of the reports on alloSCT are from Japan. Large retrospective Japanese studies and a smaller European report demonstrate that alloSCT results in long-term survival in roughly one third of transplanted patients but only a small percentage of patients can make it to transplant (Hishizawa et al., 2010; Bazarbachi et al., 2014).

Overall, current treatments of aggressive ATL subtypes are not satisfactory. Indeed, patients with acute and lymphoma subtypes who do not respond to primary therapy remain a population with unmet medical need. The lack of curative therapy of ATL, and the low survival rates in ATL patients inquire exploring new targeted therapies to improve survival and achieve cure for these patients.

## Innovative Therapies of Adult T Cell Leukemia

### Monoclonal Antibodies

#### Mogamulizumab

C-C chemokine receptor 4 is a chemokine receptor known to be selectively expressed in type 2 helper T cells (Th2 cells) and regulatory T cells (T reg) (Ishida and Ueda, 2006). CCR4 is involved in leukocyte migration and is expressed on ATL cells. Mogamulizumab (KW-0761) is a humanized defucosylated monoclonal antibody targeting CCR4 (Ishii et al., 2010; Subramaniam et al., 2012; Tobinai et al., 2012). Interestingly, Mogamulizumab exhibits its antitumor activity in ATL by various mechanisms of action. Studies have shown that this drug induces a depletion of T_*reg*_ leading to an increased antitumor immune response (Sugiyama et al., 2013; Ni et al., 2015). In addition, it highly increases antibody-dependent cellular cytotoxicity because of its reduced fucose (Shinkawa et al., 2003; Ishii et al., 2010). In Japan, this drug is approved for treatment of patients with different T cell malignancies such as relapsed/refractory (R/R) CCR4^+^ ATL and cutaneous T-cell lymphoma (CTCL) (Ishii et al., 2010). The efficacy of Mogamulizumab was tested in 28 patients with relapsed ATL (Ishida et al., 2012). The overall response rate (ORR) was 50% with 8 CR and 5 PR, and the OS was 13.7 months (Ishida et al., 2012). Similarly, Mogamulizumab showed an efficacy in Phase I study for R/R ATL and peripheral T-cell lymphoma (PTCL) in Japan with a response rate of 31% (Makita and Tobinai, 2017), and in a randomized Phase II study conducted on R/R patients in the United States and Europe (Makita and Tobinai, 2017, reviewed in Hermine et al., 2018). Mogamulizumab also improved response rate in newly diagnosed ATL patients when combined to dose-intensified chemotherapy but failed to improve progression free and overall survival (Ishida et al., 2015).

#### Anti-CD25 Antibodies

Adult T cell leukemia cells are known to express CD25, the alpha chain of the human IL-2. Thus, the efficacy of naked or Yttrium-90 anti-CD25 antibody was tested yielding few CR in indolent subtypes (Waldmann et al., 1993, 1995). Daclizumab is another humanized monoclonal antibody targeting CD25 (Berkowitz et al., 2014). In ATL, a phase 2 clinical trial demonstrated that Daclizumab blocks IL-2 binding to CD25 on ATL cells. Thus, an effective clinical response was acquired, especially in patients with indolent ATL (Berkowitz et al., 2014, reviewed in Hermine et al., 2018). This monoclonal was withdrawn from the market in March 2018.

#### Anti-transferrin Receptor Antibody

HTLV-1 infected cells were shown to constitutively express high levels of surface transferrin receptor. A24 is a monoclonal antibody directed against this receptor. Only pre-clinical studies were performed using this antibody. A24 induced apoptosis in both ATL cells and primary ATL cells from both acute and chronic ATL patients. Furthermore, A24 also inhibited (^55^Fe)-transferrin uptake in activated T cells and blocked T-cell proliferation *in vitro* and *ex vivo* (Moura et al., 2004).

#### Anti-KIR3DL2 Monoclonal Antibody

KIR3DL2 (CD158K), a killer immunoglobulin-like receptor (KIR) normally expressed by a subset of natural killer (NK) cells is aberrantly expressed in Sezary syndrome and several other CTCLs (Battistella et al., 2017). In few ATL patients, circulating tumor cells may also express KIR3DL2 (Obama et al., 2007), a simple biomarker that identifies most acute-type ATL (Cheminant et al. unpublished data). It has been suggested that HTLV-1 may play a role in KIR3DL2 expression, combined to DNA methylation of the *KIR3DL2* gene promoter. Moreover, IPH4102, a monoclonal antibody directed against KIR3DL2 that has shown beneficial clinical activity in relapsed CTCL patients (Bagot et al., 2019), can selectively kill human KIR3DL2+ primary ATL cells *ex-vivo* (Cheminant et al. unpublished data).

#### Alemtuzumab

Alemtuzumab (Campath-1H) is a chimeric humanized antibody that binds to a glycoprotein (CD52), which is highly expressed on benign and malignant B and T- lymphocytes and monocytes (Buggins et al., 2002). Alemtuzumab showed an antitumor activity in various cancer types such as T-cell prolymphocytic leukemia and B-cell chronic lymphocytic leukemia (Laribi et al., 2017). In ATL, a phase 2 clinical study using Alemtuzumab in acute, chronic, and lymphoma subtypes resulted in an ORR of 52%, mostly in the blood, but unfortunately with a short duration of response (Sharma et al., 2017, reviewed in Hermine et al., 2018).

#### Brentuximab Vedotin

Brentuximab vedotin (BV or SGN-35), is an anti-CD30 monoclonal antibody, conjugated to a microtubule poisoning agent monomethyl auristatin E. The latter is a cytotoxic agent, released by proteolytic cleavage following binding to anti-CD30 expressing cells, responsible of cell death induction (Laribi et al., 2018). Brentuximab vedotin is approved for the treatment of many tumors such as Hodgkin lymphoma (HL), CD30^+^ PTCL, and CD30^+^ CTCL. Several clinical trials are currently being conducted on patients with R/R CD30^+^ lymphomas, including some ATL patients under a pilot study in patients with R/R disease (NCT01703949), and a phase 2 study on patients with R/R CD30-low mature T-cell lymphomas (Hermine et al., 2018). Importantly, the ECHELON 2 randomized trial demonstrated that front-line treatment with BV and chemotherapy is superior to CHOP for patients with CD30-positive PTCL as shown by a significant improvement in PFS and OS with a manageable safety profile (Horwitz et al., 2019).

#### Anti-PD-1 Antibodies

Genomic analysis has shown a higher mutation rate in ATL than in other hematopoietic cancers. These changes include the T-cell receptor, nuclear factor κB, and immune surveillance pathways, including overexpression of the programmed cell death 1 ligand (PD-L1) gene. Kataoka et al. (2016) reported the genetic evidence of clonal selection of ATL cells by way of PD-L1 overexpression through disruption of its 3′ untranslated region (UTR) in 16% of their cohort. Several phase 1/2 studies of nivolumab, an anti-PD-1 antibody, are ongoing. Among them, Ratner et al. reported rapid progression of ATL in sequential three patients after the treatment (Ratner and the Authors, 2018; Ratner et al., 2018), supporting for the probable role of PD-1 functioning as a tumor suppressor reported in a mouse model (Wartewig et al., 2017). However, the other group did not experience such progression, requiring further evaluation of anti-PD-1 antibodies for especially aggressive ATL with PD-L1 overexpression (Ishitsuka et al., 2018).

### Lenalidomide

Lenalidomide, a thalidomide analog, is an immunomodulatory drug with pleiotropic mechanisms of action, involving anti-inflammatory, antiangiogenic, and antitumor effects (reviewed in Kotla et al., 2009; Ito and Handa, 2016; Chamberlain and Cathers, 2019). Lenalidomide was approved by the FDA for the treatment of multiple myeloma, mantle cell lymphoma, and myelodysplastic syndrome with deletion 5q. In addition, lenalidomide exhibited clinical activity in other malignancies including acute myeloid leukemia and non-Hodgkin lymphoma. Its immunomodulatory properties include augmentation of NK cell number and cytotoxicity, activation of T cells, and alteration of cytokine production by monocytes (Kotla et al., 2009; Ito and Handa, 2016).

Clinical trials on lenalidomide monotherapy in ATL include a phase 1 dose-escalation study on Japanese patients with ATL and PTCL (Ogura et al., 2016). Lenalidomide safety, maximum tolerated dose, and efficacy were investigated in a small cohort study of 13 patients (9 ATL, 4 PTCL). A maximum tolerated dose of 25 mg daily on each day of 28-day cycle was recommended for phase 2 studies (Ogura et al., 2016). Subsequently, a multicenter phase 2 open-label study of 26 patients with relapsed/recurrent ATL (15 acute type, 7 lymphoma type, and 4 chronic type) was conducted (Ishida et al., 2016). Lenalidomide demonstrated significant anti-leukemic activity, accompanied by a tolerable toxicity profile. Briefly, the CR and response rates were 19 and 42% respectively with a median overall survival of 20 months. This study resulted in the approval of lenalidomide for clinical use in Japan for treating R/R ATL patients in March 2017 (Ishida et al., 2016).

Recently, a case report by Oka et al. (2019) described a potential for low-dose lenalidomide in maintenance therapy of ATL. Briefly, daily treatment with low-dose lenalidomide (5 mg/day) following LSG15 chemotherapy with mogamulizumab maintained complete remission in a patient with acute ATL, whose condition remained stable with no recurrence for at least 24 months. Lenalidomide triggered an increase in the numbers of CD3^+^ CD8^+^ cytotoxic T cells, CD3^+^ CD4^+^T cells and CD56^+^ CD16^+^?NK cells. This report warrants further investigations on possible promising combination of lenalidomide with monoclonal antibodies and chemotherapy in patients who are not eligible for HSCT. In fact, given that mogamulizumab antitumor activity completely depends on antibody dependent cellular cytotoxicity, mainly through NK cells, while lenalidomide augments NK cells activity and number, investigating a combination of both drugs in ATL treatment might result in favorable outcomes.

### Arsenic Trioxide and Interferon-Alpha

Despite the important advances in the treatment of ATL using the combination of AZT/IFN many patients are either resistant or progress even after a long period of disease control. Overall, the lack of curative therapy of ATL, in patients as well as in preclinical or naturally infected models (Turpin et al., 2017), underlies the importance of exploring new therapies targeting ATL leukemia initiating cells (LICs), to achieve disease eradication, rather than long term disease control. *In vitro*, the combination of arsenic trioxide (AS) and IFN selectively induces cell cycle arrest and apoptosis of ATL cells (Bazarbachi et al., 1999). This was associated with proteasome-mediated Tax degradation (El-Sabban et al., 2000; Nasr et al., 2003). This AS/IFN combination cured Tax-driven murine ATL through LIC eradication and delayed tumor cell exhaustion (El Hajj et al., 2010). Critically, adding the proteasome inhibitor bortezomib (PS-341) essentially blocks the degradation of Tax triggered by the AS/IFN combination, and reversed the enhancement of survival in secondary and tertiary recipients in serial transplantation assays. Overall, ATL cells are addicted to continuous Tax expression for their LIC activity and long term proliferation but not for their short-term tumor growth (El Hajj et al., 2010).

A phase 2 trial involving seven patients with relapsed/refractory ATL showed that AS/IFN combination induced one complete remission and three partial remissions, with one patient who remained alive and disease free for more than 5 years (Hermine et al., 2004 and unpublished). A prospective phase 2 study tested the efficacy, and safety of the combination of AS/IFN/AZT in 10 *de novo* chronic ATL patients (Kchour et al., 2009). Strikingly, AS/IFN/AZT therapy led to a 100% response rate and 70% complete remission rate (7 CR, 2 CR with 5% circulating atypical lymphocytes and 1 PR). Some patients exhibited a sustained response, even after treatment withdrawal, suggesting a potential cure through ATL LICs loss (Kchour et al., 2009). Side effects were very moderate and mostly hematologic. Although these treated patients received a suboptimal 5-days-per-week treatment, 3 of 6 patients remained in continuous CR for 7–18 months after discontinuation of maintenance therapy, as compared to five patients with chronic ATL treated with AZT/IFN alone who all relapsed before 5 months (El Hajj et al., 2010). Recently, a clinical trial tested AS consolidation in nine patients with ATL (four patients with lymphoma, two patients with acute, and three patients with indolent ATL). Four patients were in CR, three patients were in PR, one patient in stable disease and the last one was in progressive disease. These patients received up to eight weeks of intravenous AS at the dose of 0.15 mg/kg/day intravenously in combination with AZT/IFN. One patient died rapidly, while the remaining eight patients showed prolonged median duration of response of 24 and 39 months from initiation of AS consolidation and from diagnosis, respectively (Marcais et al., 2020). These results suggest that consolidation with arsenic could be an option for ATL patients achieving a satisfactory response after induction therapy and who are not eligible for HSCT (Marcais et al., 2020).

### Vaccines

Recent efforts suggested anti-ATL vaccines as a potential therapeutic option for ATL. These vaccines are meant to stimulate host immune response against the virus, and may provide some clinical benefits, especially for R/R ATL. A Tax peptide-pulsed dendritic cell (Tax-DC) vaccine was designed to augment Tax-specific cytotoxic T lymphocyte (CTL) response. This vaccine consists of autologous DCs pulsed with different Tax peptides known to be the Tax-inducing CTL epitopes (Suehiro et al., 2015). A pilot clinical trial was performed on three previously treated ATL patients who were classified as intermediate to high risk patients (Suehiro et al., 2015), and led to favorable clinical outcomes. Indeed, two patients survived for more than 4 years after vaccination without severe adverse effects (Kannagi et al., 2019a). This Tax-DC vaccine is currently under a phase 1 trial, with a promising clinical outcome. This vaccine can represent a safe alternative maintenance therapy for ATL. It can also be applied in case of indolent ATL or might be a prophylactic approach in HTLV-1 carriers (Kannagi et al., 2019b).

Another therapeutic vaccine candidate for treatment of ATL, called THV02, comprises two lentiviral vectors to be used in a prime/boost regimen. This vaccine encodes for a peptide deriving from the viral proteins Tax, HBZ, p12I, and p30II. THV02 proved to induce a cellular response in animal models warranting conducting clinical trials to test its efficacy (reviewed in Hermine et al., 2018).

### Epigenetic Therapies Against ATL

#### Ezh Inhibitors

Enhancer of Zeste Homolog 1 and 2 (EZH1 and EZH2) are polycomb repressive protein components (PRC). EZH2, the catalytic subunit of PRC2, mediates transcriptional silencing through trimethylation of histone H3 lysine 27 (H_3_K_27_^*me3*^). Mutation or overexpression of EZH2 has been linked to numerous types of cancer and EZH2 inhibitors are being evaluated in clinical trials as potential anti-cancer agents (reviewed in Lund et al., 2014; Gan et al., 2018; Nakagawa and Kitabayashi, 2018). More recently, significance of EZH1 has been recognized also (Yamagishi et al., 2019). In ATL cells, miR-31 level is epigenetically regulated, and aberrant upregulation of polycomb proteins including EZH1/2 contribute to miR-31 downregulation in an epigenetic fashion, leading to activation of NF-κB and apoptosis resistance (Yamagishi et al., 2012). EZH2 is affected by the HTLV-1 Tax protein. Likewise, the Tax-dependent immortalized cells showed H_3_K_27_^*me3*^ reprogramming that was significantly similar to that of ATL cells (Fujikawa et al., 2016).

Pharmacologic inhibition of EZH2, *in vitro*, in ATL cells resulted in reversed epigenetic aberrations and selective elimination of leukemic and HTLV-1-infected cells presenting EZH2 as possible target for epigenetic therapy in ATL (Fujikawa et al., 2016).

Valemetostat (DS-3201) is a potent selective dual inhibitor of EZH1 and EZH2 with antineoplastic potential especially in lymphomas including ATL (Yamagishi et al., 2019). A multicenter, Phase 1 multiple ascending dose study of DS-3201b in Japanese patients with R/R NHL including ATL was conducted to evaluate the safety, pharmacokinetics, and recommended dose of DS-3201. Based on the promising results, a phase 2 trial of valemetostat for R/R aggressive ATL after mogamulizumab treatment is ongoing in Japan.

#### Histone Deacytelase Inhibitors

Histone deacetylase inhibitors (HDACI) are anti-cancer drugs that relieve histone deacetylation imposed by HDAC resulting in chromatin remodeling and reactivation of transcriptionally suppressed genes (reviewed in Li and Seto, 2016). Several HDACIs, vorinostat, romidepsin and belinostat, were FDA approved for the treatment of PTCL. These include Vorinostat (SAHA) and Romidepsin for CTCL. Vorinostat induced 30% response rates (Olsen et al., 2007), while Romidepsin induced 34% response rates (Whittaker et al., 2010). Combining valproic acid, a first generation HDACI, with AZT/IFN as a maintenance therapy was tested in 13 ATL patients (Ramos and Lossos, 2011). A decrease of clonal disease was observed by PCR, only in one patient. However, primary ATL cells from this patient showed an increased HTLV-I expression and induced apoptosis upon treatment with vorinostat. Thus, these results could be extrapolated to eliminate residual disease (Afonso et al., 2010).

*In vitro*, many HDAC inhibitors exhibited excellent anti-leukemic potential against HTLV-1 transformed or ATL-derived cell lines and primary ATL cells by blocking Notch pathway, decreasing NF-κB, and promoting apoptosis (Mori et al., 2004; Nishioka et al., 2008; Yu et al., 2015). These include valproic acid, vorinostat, romidepsin, panobinostat, and entinostat (Mori et al., 2004; Nishioka et al., 2008). An orally bioavailable HDACi, AR-42 reduced the proliferation of ATL cell lines by promoting apoptosis and histone hyperacetylation and enhanced the survival of NOD/SCID ATL mouse model (Zimmerman et al., 2011). Romidepsin as single agent prolonged survival in MT1 NOD/SCID mouse model (Yu et al., 2015). Combination of Romidepsin with anti CD25 depsipeptide resulted in prolonged survival of a Met-1 ATL murine model (Chen et al., 2009). Panobinostat, a HDACI, decreased in levels of factors involved in ATLL cell proliferation and invasion such as CCR4, IL-2R and HTLV-1 HBZ-SI, a spliced form of the HTLV-1 basic zipper factor HBZ, and induced apoptosis in ATL cells via activation of a novel RAIDD-caspase-2 pathway (Hasegawa et al., 2011).

Clinical trials on HDAC included a phase 1 trial on combination of Alisertib (an Aurora kinase A inhibitor) and vorinostat in treating relapsed/recurrent Hodgkins lymphoma, B-cell non-Hodkin lymphoma, and PTCL (Siddiqi et al., 2019). Alisertib combined to vorinostat showed encouraging clinical activity with a manageable safety profile.

Chidamide, which is an orally bioavailable member of the benzamide class of HDACI, was approved by CFDA in China for treating relapsed/refractory PTCL as monotherapy with a recommended dose of 30 mg twice weekly (BIW) although the dose finding results were not conclusive (reviewed in Zhang et al., 2019). A phase 1 trial of chidamide in Japan for patients with R/R NHL including ATL revealed that MTD was 40 mg BIW and best overall response was noted in 40 mg BIW cohort (N = 7): 1 CR, 5 PR, 1 SD. Four of the partial responders were ATL patients (among total five patients). In the 30 mg BIW dose cohort, 4/6 patients had stable disease after the 1st cycle (reviewed in Zhang et al., 2019). Based on the promising results, a phase 2 trial of chidamide for R/R aggressive ATL after mogamulizumab treatment is ongoing in Japan.

## Concluding Remarks

Adult T cell leukemia remains a challenging disease with no current satisfying therapy. Indeed, about 40% of the patients after allo-HSCT, and 10–20% of those after chemotherapy could be cured without treatment for more than 5 years. Indolent forms of the disease present a long-term survival following treatment with antiviral therapies. The aggressive forms of the disease remain an unmet medical need, with only a small number of patients who can achieve long-term survival either following CR on antiviral therapy or if they make it to intensive chemotherapy +/− allo-HSCT. Pilot studies with Tax vaccination are promising. Monoclonal antibodies alone or combined to chemotherapy improved response rates, yet with little effect, if any, on overall survival. The use of epigenetic inhibitors yielded promising preclinical and phase 1 results. Arsenic trioxide combined with IFA targets Tax for degradation and cures murine ATL through elimination of LIC activity. Clinical studies suggest that addition of arsenic to antiviral therapy, or as a consolidation therapy, may achieve disease eradication and long-term survival. Based on the progress in understanding the pathophysiology of ATL, and the risk-adapted treatment approaches to different ATL subtypes, future strategies should include new targeted therapies that take into account the host immune responses and the host microenvironment including HTLV-1 infected non-malignant cells. That might lead the prevention of ATL development among high-risk HTLV-1 carriers.

## Author Contributions

All authors contributed to the writing of this review, each under his/her area of expertise and approved this version before its submission.

## Conflict of Interest

The authors declare that the research was conducted in the absence of any commercial or financial relationships that could be construed as a potential conflict of interest.

## References

[B1] Afonso P. V. Cassar O. Gessain A. (2019). Molecular epidemiology, genetic variability and evolution of HTLV-1 with special emphasis on African genotypes. *Retrovirology* 16:39. 10.1186/s12977-019-0504-z 31842895 PMC6916231

[B2] Afonso P. V. Mekaouche M. Mortreux F. Toulza F. Moriceau A. Wattel E. (2010). Highly active antiretroviral treatment against STLV-1 infection combining reverse transcriptase and HDAC inhibitors. *Blood* 116 3802–3808. 10.1182/blood-2010-02-270751 20587783

[B3] Arnold J. Zimmerman B. Li M. Lairmore M. D. Green P. L. (2008). Human T-cell leukemia virus type-1 antisense-encoded gene, Hbz, promotes T-lymphocyte proliferation. *Blood* 112 3788–3797. 10.1182/blood-2008-04-154286 18689544 PMC2572803

[B4] Azimi N. Jacobson S. Leist T. Waldmann T. A. (1999). Involvement of IL-15 in the pathogenesis of human T lymphotropic virus type I-associated myelopathy/tropical spastic paraparesis: implications for therapy with a monoclonal antibody directed to the IL-2/15R beta receptor. *J. Immunol.* 163 4064–4072. 10491011

[B5] Bagot M. Porcu P. Marie-Cardine A. Battistella M. William B. M. Vermeer M. (2019). IPH4102, a first-in-class anti-KIR3DL2 monoclonal antibody, in patients with relapsed or refractory cutaneous T-cell lymphoma: an international, first-in-human, open-label, phase 1 trial. *Lancet Oncol.* 20 1160–1170. 10.1016/S1470-2045(19)30320-1 31253572

[B6] Bamford R. N. Grant A. J. Burton J. D. Peters C. Kurys G. Goldman C. K. (1994). The interleukin (IL) 2 receptor beta chain is shared by IL-2 and a cytokine, provisionally designated IL-T, that stimulates T-cell proliferation and the induction of lymphokine-activated killer cells. *Proc. Natl. Acad. Sci. U.S.A.* 91 4940–4944. 10.1073/pnas.91.11.4940 8197161 PMC43905

[B7] Banerjee P. Rochford R. Antel J. Canute G. Wrzesinski S. Sieburg M. (2007). Proinflammatory cytokine gene induction by human T-cell leukemia virus type 1 (HTLV-1) and HTLV-2 Tax in primary human glial cells. *J. Virol.* 81 1690–1700. 10.1128/JVI.01513-06 17121800 PMC1797548

[B8] Bangham C. R. M. (2018). Human T cell leukemia virus type 1: persistence and pathogenesis. *Annu. Rev. Immunol.* 36 43–71. 10.1146/annurev-immunol-042617-053222 29144838

[B9] Battistella M. Leboeuf C. Ram-Wolff C. Hurabielle C. Bonnafous C. Sicard H. (2017). KIR3DL2 expression in cutaneous T-cell lymphomas: expanding the spectrum for KIR3DL2 targeting. *Blood* 130 2900–2902. 10.1182/blood-2017-06-792382 29089310

[B10] Bazarbachi A. Abou Merhi R. Gessain A. Talhouk R. El-Khoury H. Nasr R. (2004a). Human T-cell lymphotropic virus type I-infected cells extravasate through the endothelial barrier by a local angiogenesis-like mechanism. *Cancer Res.* 64 2039–2046. 10.1158/0008-5472.can-03-2390 15026341

[B11] Bazarbachi A. Cwynarski K. Boumendil A. Finel H. Fields P. Raj K. (2014). Outcome of patients with HTLV-1-associated adult T-cell leukemia/lymphoma after SCT: a retrospective study by the EBMT LWP. *Bone Marrow Transplant.* 49 1266–1268. 10.1038/bmt.2014.143 25029232

[B12] Bazarbachi A. El-Sabban M. E. Nasr R. Quignon F. Awaraji C. Kersual J. (1999). Arsenic trioxide and interferon-alpha synergize to induce cell cycle arrest and apoptosis in human T-cell lymphotropic virus type I-transformed cells. *Blood* 93 278–283. 9864171

[B13] Bazarbachi A. Ghez D. Lepelletier Y. Nasr R. de The H. El-Sabban M. E. (2004b). New therapeutic approaches for adult T-cell leukaemia. *Lancet Oncol.* 5 664–672. 15522654 10.1016/S1470-2045(04)01608-0

[B14] Bazarbachi A. Hermine O. (1996). Treatment with a combination of zidovudine and alpha-interferon in naive and pretreated adult T-cell leukemia/lymphoma patients. *J. Acquir. Immune Defic. Syndr. Hum. Retrovirol.* 13(Suppl. 1), S186–S190. 8797722 10.1097/00042560-199600001-00028

[B15] Bazarbachi A. Plumelle Y. Carlos Ramos J. Tortevoye P. Otrock Z. Taylor G. (2010). Meta-analysis on the use of zidovudine and interferon-alfa in adult T-cell leukemia/lymphoma showing improved survival in the leukemic subtypes. *J. Clin. Oncol.* 28 4177–4183. 20585095 10.1200/JCO.2010.28.0669

[B16] Bazarbachi A. Suarez F. Fields P. Hermine O. (2011). How I treat adult T-cell leukemia/lymphoma. *Blood* 118 1736–1745. 10.1182/blood-2011-03-345702 21673346

[B17] Berkowitz J. L. Janik J. E. Stewart D. M. Jaffe E. S. Stetler-Stevenson M. Shih J. H. (2014). Safety, efficacy, and pharmacokinetics/pharmacodynamics of daclizumab (anti-CD25) in patients with adult T-cell leukemia/lymphoma. *Clin. Immunol.* 155 176–187. 10.1016/j.clim.2014.09.012 25267440 PMC4306230

[B18] Billman M. R. Rueda D. Bangham C. R. M. (2017). Single-cell heterogeneity and cell-cycle-related viral gene bursts in the human leukaemia virus HTLV-1. *Wellcome Open Res.* 2:87 10.12688/wellcomeopenres.12469.2 PMC5645716 29062917

[B19] Bittencourt A. L. da Gracas Vieira M. Brites C. R. Farre L. Barbosa H. S. (2007). Adult T-cell leukemia/lymphoma in Bahia, Brazil: analysis of prognostic factors in a group of 70 patients. *Am. J. Clin. Pathol.* 128 875–882. 10.1309/2YGD1P0QCVCWBLDX 17951212

[B20] Blayney D. W. Jaffe E. S. Blattner W. A. Cossman J. Robert-Guroff M. Longo D. L. (1983). The human T-cell leukemia/lymphoma virus associated with American adult T-cell leukemia/lymphoma. *Blood* 62 401–405. 6223675

[B21] Buggins A. G. Mufti G. J. Salisbury J. Codd J. Westwood N. Arno M. (2002). Peripheral blood but not tissue dendritic cells express CD52 and are depleted by treatment with alemtuzumab. *Blood* 100 1715–1720. 12176892

[B22] Bunn P. A. Jr. Schechter G. P. Jaffe E. Blayney D. Young R. C. Matthews M. J. (1983). Clinical course of retrovirus-associated adult T-cell lymphoma in the United States. *N. Engl. J. Med.* 309 257–264. 10.1056/NEJM198308043090501 6602943

[B23] Chamberlain P. P. Cathers B. E. (2019). Cereblon modulators: low molecular weight inducers of protein degradation. *Drug Discov. Today Technol.* 31 29–34. 10.1016/j.ddtec.2019.02.004 31200856

[B24] Chen J. Zhang M. Ju W. Waldmann T. A. (2009). Effective treatment of a murine model of adult T-cell leukemia using depsipeptide and its combination with unmodified daclizumab directed toward CD25. *Blood* 113 1287–1293. 10.1182/blood-2008-04-149658 18948574 PMC2637191

[B25] Chihara D. Ito H. Katanoda K. Shibata A. Matsuda T. Tajima K. (2012). Increase in incidence of adult T-cell leukemia/lymphoma in non-endemic areas of Japan and the United States. *Cancer Sci.* 103 1857–1860. 10.1111/j.1349-7006.2012.02373.x 22738276 PMC7659271

[B26] Cook L. B. Fuji S. Hermine O. Bazarbachi A. Ramos J. C. Ratner L. (2019). Revised adult T-cell leukemia-lymphoma international consensus meeting report. *J. Clin. Oncol.* 37 677–687. 10.1200/JCO.18.00501 30657736 PMC6494249

[B27] Dasanu C. A. (2011). Newer developments in adult T-cell leukemia/lymphoma therapeutics. *Expert Opin. Pharmacother.* 12 1709–1717. 10.1517/14656566.2011.571207 21486117

[B28] Dassouki Z. Sahin U. El Hajj H. Jollivet F. Kfoury Y. Lallemand-Breitenbach V. (2015). ATL response to arsenic/interferon therapy is triggered by SUMO/PML/RNF4-dependent Tax degradation. *Blood* 125 474–482. 25395419 10.1182/blood-2014-04-572750

[B29] Dearden C. Matutes E. Catovsky D. (1991). Deoxycoformycin in the treatment of mature T-cell leukaemias. *Br. J. Cancer* 64 903–906. 10.1038/bjc.1991.423 1931613 PMC1977476

[B30] El Hajj H. El-Sabban M. Hasegawa H. Zaatari G. Ablain J. Saab S. T. (2010). Therapy-induced selective loss of leukemia-initiating activity in murine adult T cell leukemia. *J. Exp. Med.* 207 2785–2792. 21135137 10.1084/jem.20101095 PMC3005222

[B31] El-Sabban M. E. Merhi R. A. Haidar H. A. Arnulf B. Khoury H. Basbous J. (2002). Human T-cell lymphotropic virus type 1-transformed cells induce angiogenesis and establish functional gap junctions with endothelial cells. *Blood* 99 3383–3389. 11964307 10.1182/blood.v99.9.3383

[B32] El-Sabban M. E. Nasr R. Dbaibo G. Hermine O. Abboushi N. Quignon F. (2000). Arsenic-interferon-alpha-triggered apoptosis in HTLV-I transformed cells is associated with tax down-regulation and reversal of NF-kappa B activation. *Blood* 96 2849–2855. 11023521

[B33] Fujikawa D. Nakagawa S. Hori M. Kurokawa N. Soejima A. Nakano K. (2016). Polycomb-dependent epigenetic landscape in adult T-cell leukemia. *Blood* 127 1790–1802. 10.1182/blood-2015-08-662593 26773042

[B34] Gan L. Yang Y. Li Q. Feng Y. Liu T. Guo W. (2018). Epigenetic regulation of cancer progression by EZH2: from biological insights to therapeutic potential. *Biomark Res.* 6:10 10.1186/s40364-018-0122-2 PMC5845366 29556394

[B35] Gaudray G. Gachon F. Basbous J. Biard-Piechaczyk M. Devaux C. Mesnard J. M. (2002). The complementary strand of the human T-cell leukemia virus type 1 RNA genome encodes a bZIP transcription factor that down-regulates viral transcription. *J. Virol.* 76 12813–12822. 10.1128/jvi.76.24.12813-12822.2002 12438606 PMC136662

[B36] Gessain A. (2011). [Human retrovirus HTLV-1: descriptive and molecular epidemiology, origin, evolution, diagnosis and associated diseases]. *Bull. Soc. Pathol. Exot.* 104 167–180. 10.1007/s13149-011-0174-4 21796326

[B37] Gessain A. Mahieux R. (2000). [Epidemiology, origin and genetic diversity of HTLV-1 retrovirus and STLV-1 simian affiliated retrovirus]. *Bull. Soc. Pathol. Exot.* 93 163–171. 11030050

[B38] Gill P. S. Harrington W. Jr. Kaplan M. H. Ribeiro R. C. Bennett J. M. Liebman H. A. (1995). Treatment of adult T-cell leukemia-lymphoma with a combination of interferon alfa and zidovudine. *N. Engl. J. Med.* 332 1744–1748. 10.1056/NEJM199506293322603 7760890

[B39] Harhaj E. W. Sun S. C. (1999). IKKgamma serves as a docking subunit of the IkappaB kinase (IKK) and mediates interaction of IKK with the human T-cell leukemia virus Tax protein. *J. Biol. Chem.* 274 22911–22914. 10.1074/jbc.274.33.22911 10438454

[B40] Hasegawa H. Sawa H. Lewis M. J. Orba Y. Sheehy N. Yamamoto Y. (2006). Thymus-derived leukemia-lymphoma in mice transgenic for the Tax gene of human T-lymphotropic virus type I. *Nat. Med.* 12 466–472. 16550188 10.1038/nm1389

[B41] Hasegawa H. Yamada Y. Tsukasaki K. Mori N. Tsuruda K. Sasaki D. (2011). LBH589, a deacetylase inhibitor, induces apoptosis in adult T-cell leukemia/lymphoma cells via activation of a novel RAIDD-caspase-2 pathway. *Leukemia* 25 575–587. 10.1038/leu.2010.315 21242994 PMC3089964

[B42] Hermine O. Allard I. Levy V. Arnulf B. Gessain A. Bazarbachi A. (2002). A prospective phase II clinical trial with the use of zidovudine and interferon-alpha in the acute and lymphoma forms of adult T-cell leukemia/lymphoma. *Hematol. J.* 3 276–282. 10.1038/sj.thj.6200195 12522449

[B43] Hermine O. Bouscary D. Gessain A. Turlure P. Leblond V. Franck N. (1995). Brief report: treatment of adult T-cell leukemia-lymphoma with zidovudine and interferon alfa. *N. Engl. J. Med.* 332 1749–1751. 10.1056/NEJM199506293322604 7760891

[B44] Hermine O. Dombret H. Poupon J. Arnulf B. Lefrere F. Rousselot P. (2004). Phase II trial of arsenic trioxide and alpha interferon in patients with relapsed/refractory adult T-cell leukemia/lymphoma. *Hematol. J.* 5 130–134. 10.1038/sj.thj.6200374 15048063

[B45] Hermine O. Ramos J. C. Tobinai K. (2018). A review of new findings in adult T-cell leukemia-lymphoma: a focus on current and emerging treatment strategies. *Adv. Ther.* 35 135–152. 10.1007/s12325-018-0658-4 29411267 PMC5818559

[B46] Hermine O. Wattel E. Gessain A. Bazarbachi A. (1998). Adult T cell leukaemia: a review of established and new treatments. *Biodrugs* 10 447–462. 10.2165/00063030-199810060-00003 18020614

[B47] Hishizawa M. Kanda J. Utsunomiya A. Taniguchi S. Eto T. Moriuchi Y. (2010). Transplantation of allogeneic hematopoietic stem cells for adult T-cell leukemia: a nationwide retrospective study. *Blood* 116 1369–1376. 10.1182/blood-2009-10-247510 20479287

[B48] Hleihel R. Khoshnood B. Dacklin I. Omran H. Mouawad C. Dassouki Z. (2018). The HTLV-1 oncoprotein Tax is modified by the ubiquitin related modifier 1 (Urm1). *Retrovirology* 15:33. 10.1186/s12977-018-0415-4 29665857 PMC5904992

[B49] Hodson A. Crichton S. Montoto S. Mir N. Matutes E. Cwynarski K. (2011). Use of zidovudine and interferon alfa with chemotherapy improves survival in both acute and lymphoma subtypes of adult T-cell leukemia/lymphoma. *J. Clin. Oncol.* 29 4696–4701. 10.1200/JCO.2011.35.5578 22042945

[B50] Horwitz S. O’Connor O. A. Pro B. Illidge T. Fanale M. Advani R. (2019). Brentuximab vedotin with chemotherapy for CD30-positive peripheral T-cell lymphoma (ECHELON-2): a global, double-blind, randomised, phase 3 trial. *Lancet* 393 229–240. 10.1016/S0140-6736(18)32984-2 30522922 PMC6436818

[B51] Iqbal M. Reljic T. Klocksieben F. Sher T. Ayala E. Murthy H. (2019). Efficacy of allogeneic hematopoietic cell transplantation in human T cell lymphotropic virus type 1-associated adult T cell leukemia/lymphoma: results of a systematic review/meta-analysis. *Biol. Blood Marrow Transplant.* 25 1695–1700. 10.1016/j.bbmt.2019.05.027 31132453

[B52] Ishida T. Fujiwara H. Nosaka K. Taira N. Abe Y. Imaizumi Y. (2016). Multicenter phase II study of lenalidomide in relapsed or recurrent adult T-cell leukemia/lymphoma: ATLL-002. *J. Clin. Oncol.* 34 4086–4093. 10.1200/JCO.2016.67.7732 27621400

[B53] Ishida T. Jo T. Takemoto S. Suzushima H. Uozumi K. Yamamoto K. (2015). Dose-intensified chemotherapy alone or in combination with mogamulizumab in newly diagnosed aggressive adult T-cell leukaemia-lymphoma: a randomized phase II study. *Br. J. Haematol.* 169 672–682. 10.1111/bjh.13338 25733162 PMC5024033

[B54] Ishida T. Joh T. Uike N. Yamamoto K. Utsunomiya A. Yoshida S. (2012). Defucosylated anti-CCR4 monoclonal antibody (KW-0761) for relapsed adult T-cell leukemia-lymphoma: a multicenter phase II study. *J. Clin. Oncol.* 30 837–842. 10.1200/JCO.2011.37.3472 22312108

[B55] Ishida T. Ueda R. (2006). CCR4 as a novel molecular target for immunotherapy of cancer. *Cancer Sci.* 97 1139–1146. 10.1111/j.1349-7006.2006.00307.x 16952304 PMC11159356

[B56] Ishii T. Ishida T. Utsunomiya A. Inagaki A. Yano H. Komatsu H. (2010). Defucosylated humanized anti-CCR4 monoclonal antibody KW-0761 as a novel immunotherapeutic agent for adult T-cell leukemia/lymphoma. *Clin. Cancer Res.* 16 1520–1531. 10.1158/1078-0432.CCR-09-2697 20160057

[B57] Ishitsuka K. Tamura K. (2014). Human T-cell leukaemia virus type I and adult T-cell leukaemia-lymphoma. *Lancet Oncol.* 15 e517–e526. 10.1016/S1470-2045(14)70202-5 25281470

[B58] Ishitsuka K. Utsunomiya A. Ishida T. (2018). PD-1 Inhibitor Therapy in Adult T-Cell Leukemia-Lymphoma. *N. Engl. J. Med.* 379:695 10.1056/NEJMc1807852 30124278

[B59] Ito T. Handa H. (2016). Cereblon and its downstream substrates as molecular targets of immunomodulatory drugs. *Int. J. Hematol.* 104 293–299. 10.1007/s12185-016-2073-4 27460676

[B60] Kannagi M. Hasegawa A. Nagano Y. Iino T. Okamura J. Suehiro Y. (2019a). Maintenance of long remission in adult T-cell leukemia by Tax-targeted vaccine: a hope for disease-preventive therapy. *Cancer Sci.* 110 849–857. 10.1111/cas.13948 30666755 PMC6398881

[B61] Kannagi M. Hasegawa A. Nagano Y. Kimpara S. Suehiro Y. (2019b). Impact of host immunity on HTLV-1 pathogenesis: potential of Tax-targeted immunotherapy against ATL. *Retrovirology* 16:23 10.1186/s12977-019-0484-z PMC6704564 31438973

[B62] Kataoka K. Shiraishi Y. Takeda Y. Sakata S. Matsumoto M. Nagano S. (2016). Aberrant PD-L1 expression through 3’-UTR disruption in multiple cancers. *Nature* 534 402–406. 10.1038/nature18294 27281199

[B63] Katsuya H. Ishitsuka K. Utsunomiya A. Hanada S. Eto T. Moriuchi Y. (2015). Treatment and survival among 1594 patients with ATL. *Blood* 126 2570–2577. 10.1182/blood-2015-03-632489 26361794

[B64] Kchour G. Tarhini M. Kooshyar M. M. El Hajj H. Wattel E. Mahmoudi M. (2009). Phase 2 study of the efficacy and safety of the combination of arsenic trioxide, interferon alpha, and zidovudine in newly diagnosed chronic adult T-cell leukemia/lymphoma (ATL). *Blood* 113 6528–6532. 19411628 10.1182/blood-2009-03-211821

[B65] Kfoury Y. Nasr R. Favre-Bonvin A. El-Sabban M. Renault N. Giron M. L. (2008). Ubiquitylated Tax targets and binds the IKK signalosome at the centrosome. *Oncogene* 27 1665–1676. 10.1038/sj.onc.1210804 17891179

[B66] Kfoury Y. Nasr R. Hermine O. de The H. Bazarbachi A. (2005). Proapoptotic regimes for HTLV-I-transformed cells: targeting Tax and the NF-kappaB pathway. *Cell Death Differ.* 12(Suppl. 1), 871–877. 15846376 10.1038/sj.cdd.4401624

[B67] Kfoury Y. Nasr R. Journo C. Mahieux R. Pique C. Bazarbachi A. (2012). The multifaceted oncoprotein Tax: subcellular localization, posttranslational modifications, and NF-kappaB activation. *Adv. Cancer Res.* 113 85–120. 10.1016/B978-0-12-394280-7.00003-8 22429853

[B68] Kfoury Y. Setterblad N. El-Sabban M. Zamborlini A. Dassouki Z. El Hajj H. (2011). Tax ubiquitylation and SUMOylation control the dynamic shuttling of Tax and NEMO between Ubc9 nuclear bodies and the centrosome. *Blood* 117 190–199. 10.1182/blood-2010-05-285742 20959607

[B69] Kinpara S. Kijiyama M. Takamori A. Hasegawa A. Sasada A. Masuda T. (2013). Interferon-alpha (IFN-alpha) suppresses HTLV-1 gene expression and cell cycling, while IFN-alpha combined with zidovudine induces p53 signaling and apoptosis in HTLV-1-infected cells. *Retrovirology* 10:52. 10.1186/1742-4690-10-52 23688327 PMC3698133

[B70] Kiyokawa T. Yamaguchi K. Takeya M. Takahashi K. Watanabe T. Matsumoto T. (1987). Hypercalcemia and osteoclast proliferation in adult T-cell leukemia. *Cancer* 59 1187–1191. 2880656 10.1002/1097-0142(19870315)59:6<1187::aid-cncr2820590626>3.0.co;2-8

[B71] Kotla V. Goel S. Nischal S. Heuck C. Vivek K. Das B. (2009). Mechanism of action of lenalidomide in hematological malignancies. *J. Hematol. Oncol.* 2:36 10.1186/1756-8722-2-36 PMC2736171 19674465

[B72] Laperche S. Worms B. Pillonel J. European Network of Transfusion Medecine Societies and Steering Committee (2009). Blood safety strategies for human T-cell lymphotropic virus in Europe. *Vox Sang* 96 104–110. 10.1111/j.1423-0410.2008.01136.x 19076337

[B73] Laribi K. Alani M. Truong C. Baugier de Materre A. (2018). Recent Advances in the Treatment of Peripheral T-Cell Lymphoma. *Oncologist* 23 1039–1053. 10.1634/theoncologist.2017-0524 29674443 PMC6192612

[B74] Laribi K. Lemaire P. Sandrini J. Baugier de Materre A. (2017). Advances in the understanding and management of T-cell prolymphocytic leukemia. *Oncotarget* 8 104664–104686. 10.18632/oncotarget.22272 29262669 PMC5732835

[B75] Larocca D. Chao L. A. Seto M. H. Brunck T. K. (1989). Human T-cell leukemia virus minus strand transcription in infected T-cells. *Biochem. Biophys. Res. Commun.* 163 1006–1013. 10.1016/0006-291x(89)92322-x 2476979

[B76] Li Y. Seto E. (2016). HDACs and HDAC inhibitors in cancer development and therapy. *Cold Spring Harb. Perspect. Med.* 6:a026831 10.1101/cshperspect.a026831 PMC5046688 27599530

[B77] Lund K. Adams P. D. Copland M. (2014). EZH2 in normal and malignant hematopoiesis. *Leukemia* 28 44–49. 10.1038/leu.2013.288 24097338

[B78] Ma G. Yasunaga J. Matsuoka M. (2016). Multifaceted functions and roles of HBZ in HTLV-1 pathogenesis. *Retrovirology* 13:16 10.1186/s12977-016-0249-x PMC4793531 26979059

[B79] Macchi B. Balestrieri E. Frezza C. Grelli S. Valletta E. Marcais A. (2017). Quantification of HTLV-1 reverse transcriptase activity in ATL patients treated with zidovudine and interferon-alpha. *Blood Adv.* 1 748–752. 10.1182/bloodadvances.2016001370 29296718 PMC5728049

[B80] Mahgoub M. Yasunaga J. I. Iwami S. Nakaoka S. Koizumi Y. Shimura K. (2018). Sporadic on/off switching of HTLV-1 Tax expression is crucial to maintain the whole population of virus-induced leukemic cells. *Proc. Natl. Acad. Sci. U.S.A.* 115 E1269–E1278. 29358408 10.1073/pnas.1715724115 PMC5819419

[B81] Mahieux R. (2015). A vaccine against HTLV-1 HBZ makes sense. *Blood* 126 1052–1053. 10.1182/blood-2015-06-652040 26316613

[B82] Makita S. Tobinai K. (2017). Mogamulizumab for the treatment of T-cell lymphoma. *Expert Opin. Biol. Ther.* 17 1145–1153. 10.1080/14712598.2017.1347634 28649848

[B83] Malpica L. Pimentel A. Reis I. M. Gotuzzo E. Lekakis L. Komanduri K. (2018). Epidemiology, clinical features, and outcome of HTLV-1-related ATLL in an area of prevalence in the United States. *Blood Adv.* 2 607–620. 29545256 10.1182/bloodadvances.2017011106 PMC5873228

[B84] Marcais A. Cook L. Witkover A. Asnafi V. Avettand-Fenoel V. Delarue R. (2020). Arsenic trioxide (As2O3) as a maintenance therapy for adult T cell leukemia/lymphoma. *Retrovirology* 17:5. 10.1186/s12977-020-0513-y 32199462 PMC7085150

[B85] Matsuoka M. Green P. L. (2009). The HBZ gene, a key player in HTLV-1 pathogenesis. *Retrovirology* 6:71 10.1186/1742-4690-6-71 PMC2731725 19650892

[B86] Matsuoka M. Jeang K. T. (2011). Human T-cell leukemia virus type 1 (HTLV-1) and leukemic transformation: viral infectivity, Tax, HBZ and therapy. *Oncogene* 30 1379–1389. 21119600 10.1038/onc.2010.537 PMC3413891

[B87] Matutes E. Taylor G. P. Cavenagh J. Pagliuca A. Bareford D. Domingo A. (2001). Interferon alpha and zidovudine therapy in adult T-cell leukaemia lymphoma: response and outcome in 15 patients. *Br. J. Haematol.* 113 779–784. 11380470 10.1046/j.1365-2141.2001.02794.x

[B88] Moodad S. Akkouche A. Hleihel R. Darwiche N. El-Sabban M. Bazarbachi A. (2018). Mouse models that enhanced our understanding of adult T cell leukemia. *Front. Microbiol.* 9:558 10.3389/fmicb.2018.00558 PMC5882783 29643841

[B89] Mori N. Matsuda T. Tadano M. Kinjo T. Yamada Y. Tsukasaki K. (2004). Apoptosis induced by the histone deacetylase inhibitor FR901228 in human T-cell leukemia virus type 1-infected T-cell lines and primary adult T-cell leukemia cells. *J. Virol.* 78 4582–4590. 10.1128/jvi.78.9.4582-4590.2004 15078940 PMC387669

[B90] Mortreux F. Kazanji M. Gabet A. S. de Thoisy B. Wattel E. (2001). Two-step nature of human T-cell leukemia virus type 1 replication in experimentally infected squirrel monkeys (Saimiri sciureus). *J. Virol.* 75 1083–1089. 10.1128/JVI.75.2.1083-1089.2001 11134325 PMC114008

[B91] Moura I. C. Lepelletier Y. Arnulf B. England P. Baude C. Beaumont C. (2004). A neutralizing monoclonal antibody (mAb A24) directed against the transferrin receptor induces apoptosis of tumor T lymphocytes from ATL patients. *Blood* 103 1838–1845. 10.1182/blood-2003-07-2440 14592824

[B92] Mulloy J. C. Kislyakova T. Cereseto A. Casareto L. LoMonico A. Fullen J. (1998). Human T-cell lymphotropic/leukemia virus type 1 Tax abrogates p53-induced cell cycle arrest and apoptosis through its CREB/ATF functional domain. *J. Virol.* 72 8852–8860. 9765430 10.1128/jvi.72.11.8852-8860.1998 PMC110302

[B93] Nakagawa M. Kitabayashi I. (2018). Oncogenic roles of enhancer of zeste homolog 1/2 in hematological malignancies. *Cancer Sci.* 109 2342–2348. 10.1111/cas.13655 29845708 PMC6113435

[B94] Nasr R. Chiari E. El-Sabban M. Mahieux R. Kfoury Y. Abdulhay M. (2006). Tax ubiquitylation and sumoylation control critical cytoplasmic and nuclear steps of NF-kappaB activation. *Blood* 107 4021–4029. 10.1182/blood-2005-09-3572 16424386

[B95] Nasr R. Rosenwald A. El-Sabban M. E. Arnulf B. Zalloua P. Lepelletier Y. (2003). Arsenic/interferon specifically reverses 2 distinct gene networks critical for the survival of HTLV-1-infected leukemic cells. *Blood* 101 4576–4582. 10.1182/blood-2002-09-2986 12560223

[B96] Ni X. Goswami M. Challagundla P. Decker W. K. Kim Y. H. Duvic M. A. (2015). Reduction of regulatory T cells by Mogamulizumab, a defucosylated anti-CC chemokine receptor 4 antibody, in patients with aggressive/refractory mycosis fungoides and Sezary syndrome. *Clin. Cancer Res.* 21 274–285. 10.1158/1078-0432.CCR-14-0830 25376389

[B97] Nishioka C. Ikezoe T. Yang J. Komatsu N. Bandobashi K. Taniguchi A. (2008). Histone deacetylase inhibitors induce growth arrest and apoptosis of HTLV-1-infected T-cells via blockade of signaling by nuclear factor kappaB. *Leuk. Res.* 32 287–296. 10.1016/j.leukres.2007.05.026 17644177

[B98] Obama K. Kubota R. Tara M. Furukawa Y. Osame M. Arimura K. (2007). Killer cell immunoglobulin-like receptor/3DL2 expression in adult T-cell leukaemia. *Br. J. Haematol.* 138 666–667. 10.1111/j.1365-2141.2007.06704.x 17686060

[B99] Ogura M. Imaizumi Y. Uike N. Asou N. Utsunomiya A. Uchida T. (2016). Lenalidomide in relapsed adult T-cell leukaemia-lymphoma or peripheral T-cell lymphoma (ATLL-001): a phase 1, multicentre, dose-escalation study. *Lancet Haematol.* 3 e107–e118. 10.1016/S2352-3026(15)00284-7 26947199

[B100] Ohsugi T. Kumasaka T. Okada S. Urano T. (2007). The Tax protein of HTLV-1 promotes oncogenesis in not only immature T cells but also mature T cells. *Nat. Med.* 13 527–528. 10.1038/nm0507-527 17479090

[B101] Oka S. Ono K. Nohgawa M. (2019). Effective maintenance treatment with lenalidomide for a patient with aggressive adult T cell leukemia after chemotherapy. *Leuk. Res. Rep.* 11 21–23. 10.1016/j.lrr.2019.04.001 31016134 PMC6475659

[B102] Olsen E. A. Kim Y. H. Kuzel T. M. Pacheco T. R. Foss F. M. Parker S. (2007). Phase IIb multicenter trial of vorinostat in patients with persistent, progressive, or treatment refractory cutaneous T-cell lymphoma. *J. Clin. Oncol.* 25 3109–3115. 10.1200/JCO.2006.10.2434 17577020

[B103] Panfil A. R. Dissinger N. J. Howard C. M. Murphy B. M. Landes K. Fernandez S. A. (2016). Functional comparison of HBZ and the related APH-2 protein provides insight into human T-cell leukemia virus type 1 pathogenesis. *J. Virol.* 90 3760–3772. 10.1128/JVI.03113-15 26819304 PMC4794683

[B104] Poiesz B. J. Ruscetti F. W. Reitz M. S. Kalyanaraman V. S. Gallo R. C. (1981). Isolation of a new type C retrovirus (HTLV) in primary uncultured cells of a patient with Sezary T-cell leukaemia. *Nature* 294 268–271. 6272125 10.1038/294268a0

[B105] Pombo De Oliveira M. S. Loureiro P. Bittencourt A. Chiattone C. Borducchi D. De Carvalho S. M. (1999). Geographic diversity of adult t-cell leukemia/lymphoma in Brazil. The Brazilian ATLL Study Group. *Int. J. Cancer* 83 291–298. 10495418 10.1002/(sici)1097-0215(19991029)83:3<291::aid-ijc1>3.0.co;2-p

[B106] Portis T. Harding J. C. Ratner L. (2001). The contribution of NF-kappa B activity to spontaneous proliferation and resistance to apoptosis in human T-cell leukemia virus type 1 Tax-induced tumors. *Blood* 98 1200–1208. 10.1182/blood.v98.4.1200 11493471

[B107] Ramos J. C. Lossos I. S. (2011). Newly emerging therapies targeting viral-related lymphomas. *Curr. Oncol. Rep.* 13 416–426. 10.1007/s11912-011-0186-8 21792540 PMC4041592

[B108] Ratner L. Harrington W. Feng X. Grant C. Jacobson S. Noy A. (2009). Human T cell leukemia virus reactivation with progression of adult T-cell leukemia-lymphoma. *PLoS One* 4:e4420. 10.1371/journal.pone.0004420 19204798 PMC2636875

[B109] Ratner L. the Authors. (2018). PD-1 inhibitor therapy in adult T-cell leukemia-lymphoma. *N. Engl. J. Med.* 379 696–697. 10.1056/NEJMc1807852 PMC9191750 30110590

[B110] Ratner L. Waldmann T. A. Janakiram M. Brammer J. E. (2018). Rapid progression of adult T-cell leukemia-lymphoma after PD-1 inhibitor therapy. *N. Engl. J. Med.* 378 1947–1948. 10.1056/NEJMc1803181 29768155

[B111] Saito M. Matsuzaki T. Satou Y. Yasunaga J. Saito K. Arimura K. (2009). *In vivo* expression of the HBZ gene of HTLV-1 correlates with proviral load, inflammatory markers and disease severity in HTLV-1 associated myelopathy/tropical spastic paraparesis (HAM/TSP). *Retrovirology* 6:19. 10.1186/1742-4690-6-19 19228429 PMC2653460

[B112] Satou Y. Yasunaga J. Yoshida M. Matsuoka M. (2006). HTLV-I basic leucine zipper factor gene mRNA supports proliferation of adult T cell leukemia cells. *Proc. Natl. Acad. Sci. U.S.A.* 103 720–725. 10.1073/pnas.0507631103 16407133 PMC1334651

[B113] Sharma K. Janik J. E. O. Mahony D. Stewart D. Pittaluga S. Stetler-Stevenson M. (2017). Phase II Study of Alemtuzumab (CAMPATH-1) in Patients with HTLV-1-Associated Adult T-cell Leukemia/lymphoma. *Clin. Cancer Res.* 23 35–42. 10.1158/1078-0432.CCR-16-1022 27486175 PMC5215752

[B114] Shimoyama M. (1991). Diagnostic criteria and classification of clinical subtypes of adult T-cell leukaemia-lymphoma. A report from the Lymphoma Study Group (1984-87). *Br. J. Haematol.* 79 428–437. 10.1111/j.1365-2141.1991.tb08051.x 1751370

[B115] Shinkawa T. Nakamura K. Yamane N. Shoji-Hosaka E. Kanda Y. Sakurada M. (2003). The absence of fucose but not the presence of galactose or bisecting N-acetylglucosamine of human IgG1 complex-type oligosaccharides shows the critical role of enhancing antibody-dependent cellular cytotoxicity. *J. Biol. Chem.* 278 3466–3473. 10.1074/jbc.M210665200 12427744

[B116] Shirinian M. Kambris Z. Hamadeh L. Grabbe C. Journo C. Mahieux R. (2015). A transgenic drosophila melanogaster model to study human T-lymphotropic virus oncoprotein tax-1-driven transformation *in vivo*. *J. Virol.* 89 8092–8095. 10.1128/JVI.00918-15 25995252 PMC4505646

[B117] Shirono K. Hattori T. Hata H. Nishimura H. Takatsuki K. (1989). Profiles of expression of activated cell antigens on peripheral blood and lymph node cells from different clinical stages of adult T-cell leukemia. *Blood* 73 1664–1671. 2713499

[B118] Siddiqi T. Frankel P. Beumer J. H. Kiesel B. F. Christner S. Ruel C. (2019). Phase 1 study of the Aurora kinase A inhibitor alisertib (MLN8237) combined with the histone deacetylase inhibitor vorinostat in lymphoid malignancies. *Leuk. Lymphoma* 61 309–317. 10.1080/10428194.2019.1672052 31617432 PMC6982547

[B119] Subramaniam J. M. Whiteside G. McKeage K. Croxtall J. C. (2012). Mogamulizumab: first global approval. *Drugs* 72 1293–1298. 10.2165/11631090-000000000-00000 22686619

[B120] Suehiro Y. Hasegawa A. Iino T. Sasada A. Watanabe N. Matsuoka M. (2015). Clinical outcomes of a novel therapeutic vaccine with Tax peptide-pulsed dendritic cells for adult T cell leukaemia/lymphoma in a pilot study. *Br. J. Haematol.* 169 356–367. 10.1111/bjh.13302 25612920

[B121] Sugata K. Yasunaga J. Mitobe Y. Miura M. Miyazato P. Kohara M. (2015). Protective effect of cytotoxic T lymphocytes targeting HTLV-1 bZIP factor. *Blood* 126 1095–1105. 10.1182/blood-2015-04-641118 26063164

[B122] Sugiyama D. Nishikawa H. Maeda Y. Nishioka M. Tanemura A. Katayama I. (2013). Anti-CCR4 mAb selectively depletes effector-type FoxP3+CD4+ regulatory T cells, evoking antitumor immune responses in humans. *Proc. Natl. Acad. Sci. U.S.A.* 110 17945–17950. 10.1073/pnas.1316796110 24127572 PMC3816454

[B123] Taguchi H. Kinoshita K. I. Takatsuki K. Tomonaga M. Araki K. Arima N. (1996). An intensive chemotherapy of adult T-cell leukemia/lymphoma: CHOP followed by etoposide, vindesine, ranimustine, and mitoxantrone with granulocyte colony-stimulating factor support. *J. Acquir Immune Defic. Syndr. Hum. Retrovirol.* 12 182–186. 10.1097/00042560-199606010-00012 8680890

[B124] Takasaki Y. Iwanaga M. Imaizumi Y. Tawara M. Joh T. Kohno T. (2010). Long-term study of indolent adult T-cell leukemia-lymphoma. *Blood* 115 4337–4343. 10.1182/blood-2009-09-242347 20348391

[B125] Takatsuki K. Uchiyama T. Sagawa K. Yodoi J. (1976). [Surface markers of malignant lymphoid cells in the classification of lymphoproliferative disorders, with special reference to adult T-cell leukemia (author’s transl)]. *Rinsho Ketsueki* 17 416–421. 1085377

[B126] Tamura K. Nagamine N. Araki Y. Seita M. Okayama A. Kawano K. (1986). Clinical analysis of 33 patients with adult T-cell leukemia (ATL)-diagnostic criteria and significance of high- and low-risk ATL. *Int. J. Cancer* 37 335–341. 10.1002/ijc.2910370303 3005175

[B127] Tanabe K. Ikegami Y. Ishida R. Andoh T. (1991). Inhibition of topoisomerase II by antitumor agents bis(2,6-dioxopiperazine) derivatives. *Cancer Res.* 51 4903–4908. 1654204

[B128] Tian Y. Kobayashi S. Ohno N. Isobe M. Tsuda M. Zaike Y. (2011). Leukemic T cells are specifically enriched in a unique CD3(dim) CD7(low) subpopulation of CD4(+) T cells in acute-type adult T-cell leukemia. *Cancer Sci.* 102 569–577. 10.1111/j.1349-7006.2010.01833.x 21205081

[B129] Tobinai K. (2009). Current management of adult T-cell leukemia/lymphoma. *Oncology* 23 1250–1256. 20120837

[B130] Tobinai K. Shimoyama M. Inoue S. Takayasu S. Mikuni C. Kozuru M. (1992). Phase I study of YK-176 (2′-deoxycoformycin) in patients with adult T-cell leukemia-lymphoma. The DCF study group. *Jpn. J. Clin. Oncol.* 22 164–171. 1518164

[B131] Tobinai K. Takahashi T. Akinaga S. (2012). Targeting chemokine receptor CCR4 in adult T-cell leukemia-lymphoma and other T-cell lymphomas. *Curr. Hematol. Malig. Rep.* 7 235–240. 10.1007/s11899-012-0124-3 22538464 PMC3425744

[B132] Tsuda H. Takatsuki K. Ohno R. Masaoka T. Okada K. Shirakawa S. (1994). Treatment of adult T-cell leukaemia-lymphoma with irinotecan hydrochloride (CPT-11), CPT-11 Study Group on Hematological Malignancy. *Br. J. Cancer* 70 771–774. 10.1038/bjc.1994.394 7917938 PMC2033383

[B133] Tsukasaki K. Hermine O. Bazarbachi A. Ratner L. Ramos J. C. Harrington W. O. Jr. (2009). Definition, prognostic factors, treatment, and response criteria of adult T-cell leukemia-lymphoma: a proposal from an international consensus meeting. *J. Clin. Oncol.* 27 453–459. 10.1200/JCO.2008.18.2428 19064971 PMC2737379

[B134] Tsukasaki K. Tobinai K. Shimoyama M. Kozuru M. Uike N. Yamada Y. (2003). Deoxycoformycin-containing combination chemotherapy for adult T-cell leukemia-lymphoma: Japan Clinical Oncology Group Study (JCOG9109). *Int. J. Hematol.* 77 164–170. 10.1007/bf02983215 12627852

[B135] Tsukasaki K. Utsunomiya A. Fukuda H. Shibata T. Fukushima T. Takatsuka Y. (2007). VCAP-AMP-VECP compared with biweekly CHOP for adult T-cell leukemia-lymphoma: Japan Clinical Oncology Group Study JCOG9801. *J. Clin. Oncol.* 25 5458–5464. 10.1200/JCO.2007.11.9958 17968021

[B136] Turpin J. Alais S. Marcais A. Bruneau J. Melamed A. Gadot N. (2017). Whole body clonality analysis in an aggressive STLV-1 associated leukemia (ATLL) reveals an unexpected clonal complexity. *Cancer Lett.* 389 78–85. 28034804 10.1016/j.canlet.2016.12.022

[B137] Uchiyama T. Hattori T. Wano Y. Tsudo M. Takatsuki K. Uchino H. (1983). Cell surface phenotype and in vitro function of adult T-cell leukemia cells. *Diagn. Immunol.* 1 150–154. 6094084

[B138] Ueda N. Iwata K. Tokuoka H. Akagi T. Ito J. Mizushima M. (1979). Adult T-cell leukemia with generalized cytomegalic inclusion disease and pneumocystis carinii pneumonia. *Acta Pathol. Jpn.* 29 221–232. 10.1111/j.1440-1827.1979.tb03176.x 233285

[B139] Verdonck K. Gonzalez E. Van Dooren S. Vandamme A. M. Vanham G. Gotuzzo E. (2007). Human T-lymphotropic virus 1: recent knowledge about an ancient infection. *Lancet Infect. Dis.* 7 266–281. 10.1016/S1473-3099(07)70081-6 17376384

[B140] Waldmann T. A. Greene W. C. Sarin P. S. Saxinger C. Blayney D. W. Blattner W. A. (1984). Functional and phenotypic comparison of human T cell leukemia/lymphoma virus positive adult T cell leukemia with human T cell leukemia/lymphoma virus negative Sezary leukemia, and their distinction using anti-Tac. Monoclonal antibody identifying the human receptor for T cell growth factor. *J. Clin. Invest.* 73 1711–1718. 10.1172/JCI111379 6327770 PMC437083

[B141] Waldmann T. A. White J. D. Carrasquillo J. A. Reynolds J. C. Paik C. H. Gansow O. A. (1995). Radioimmunotherapy of interleukin-2R alpha-expressing adult T-cell leukemia with Yttrium-90-labeled anti-Tac. *Blood* 86 4063–4075. 7492762

[B142] Waldmann T. A. White J. D. Goldman C. K. Top L. Grant A. Bamford R. (1993). The interleukin-2 receptor: a target for monoclonal antibody treatment of human T-cell lymphotrophic virus I-induced adult T-cell leukemia. *Blood* 82 1701–1712. 8400227

[B143] Wang C. Long W. Peng C. Hu L. Zhang Q. Wu A. (2016). HTLV-1 Tax Functions as a Ubiquitin E3 Ligase for Direct IKK Activation via Synthesis of Mixed-Linkage Polyubiquitin Chains. *PLoS Pathog.* 12:e1005584. 10.1371/journal.ppat.1005584 27082114 PMC4833305

[B144] Wartewig T. Kurgyis Z. Keppler S. Pechloff K. Hameister E. Ollinger R. (2017). PD-1 is a haploinsufficient suppressor of T cell lymphomagenesis. *Nature* 552 121–125. 10.1038/nature24649 29143824 PMC5821214

[B145] Watanabe T. (2017). Adult T-cell leukemia: molecular basis for clonal expansion and transformation of HTLV-1-infected T cells. *Blood* 129 1071–1081. 10.1182/blood-2016-09-692574 28115366 PMC5374731

[B146] Wattel E. Vartanian J. P. Pannetier C. Wain-Hobson S. (1995). Clonal expansion of human T-cell leukemia virus type I-infected cells in asymptomatic and symptomatic carriers without malignancy. *J. Virol.* 69 2863–2868. 7707509 10.1128/jvi.69.5.2863-2868.1995 PMC188982

[B147] White J. D. Wharfe G. Stewart D. M. Maher V. E. Eicher D. Herring B. (2001). The combination of zidovudine and interferon alpha-2B in the treatment of adult T-cell leukemia/lymphoma. *Leuk. Lymphoma* 40 287–294. 10.3109/10428190109057927 11426550

[B148] Whittaker S. J. Demierre M. F. Kim E. J. Rook A. H. Lerner A. Duvic M. (2010). Final results from a multicenter, international, pivotal study of romidepsin in refractory cutaneous T-cell lymphoma. *J. Clin. Oncol.* 28 4485–4491. 10.1200/JCO.2010.28.9066 20697094

[B149] Yamada Y. Tomonaga M. Fukuda H. Hanada S. Utsunomiya A. Tara M. (2001). A new G-CSF-supported combination chemotherapy, LSG15, for adult T-cell leukaemia-lymphoma: Japan Clinical Oncology Group Study 9303. *Br. J. Haematol.* 113 375–382. 10.1046/j.1365-2141.2001.02737.x 11380402

[B150] Yamagishi M. Hori M. Fujikawa D. Ohsugi T. Honma D. Adachi N. (2019). Targeting excessive EZH1 and EZH2 activities for abnormal histone methylation and transcription network in malignant lymphomas. *Cell Rep.* 29 2321–2337.e7. 10.1016/j.celrep.2019.10.083 31747604

[B151] Yamagishi M. Nakano K. Miyake A. Yamochi T. Kagami Y. Tsutsumi A. (2012). Polycomb-mediated loss of miR-31 activates NIK-dependent NF-kappaB pathway in adult T cell leukemia and other cancers. *Cancer Cell* 21 121–135. 10.1016/j.ccr.2011.12.015 22264793

[B152] Yamagishi M. Watanabe T. (2012). [miRNA in HTLV-1 related disease]. *Uirusu* 62 9–18. 10.2222/jsv.62.9 23189820

[B153] Yamaguchi K. Nishimura H. Kohrogi H. Jono M. Miyamoto Y. Takatsuki K. (1983). A proposal for smoldering adult T-cell leukemia: a clinicopathologic study of five cases. *Blood* 62 758–766. 6224522

[B154] Yamaoka S. Courtois G. Bessia C. Whiteside S. T. Weil R. Agou F. (1998). Complementation cloning of NEMO, a component of the IkappaB kinase complex essential for NF-kappaB activation. *Cell* 93 1231–1240. 10.1016/s0092-8674(00)81466-x 9657155

[B155] Yoshida M. Satou Y. Yasunaga J. Fujisawa J. Matsuoka M. (2008). Transcriptional control of spliced and unspliced human T-cell leukemia virus type 1 bZIP factor (HBZ) gene. *J. Virol.* 82 9359–9368. 10.1128/JVI.00242-08 18653454 PMC2546946

[B156] Yu P. Petrus M. N. Ju W. Zhang M. Conlon K. C. Nakagawa M. (2015). Augmented efficacy with the combination of blockade of the Notch-1 pathway, bortezomib and romidepsin in a murine MT-1 adult T-cell leukemia model. *Leukemia* 29 556–566. 10.1038/leu.2014.241 25118879 PMC4329116

[B157] Zhang Q. Wang S. Chen J. Yu Z. (2019). Histone Deacetylases (HDACs) Guided Novel Therapies for T-cell lymphomas. *Int. J. Med. Sci.* 16 424–442. 10.7150/ijms.30154 30911277 PMC6428980

[B158] Zhao T. Matsuoka M. (2012). HBZ and its roles in HTLV-1 oncogenesis. *Front. Microbiol.* 3:247 10.3389/fmicb.2012.00247 PMC3391691 22787458

[B159] Zimmerman B. Sargeant A. Landes K. Fernandez S. A. Chen C. S. Lairmore M. D. (2011). Efficacy of novel histone deacetylase inhibitor, AR42, in a mouse model of, human T-lymphotropic virus type 1 adult T cell lymphoma. *Leuk. Res.* 35 1491–1497. 10.1016/j.leukres.2011.07.015 21802726 PMC3191315

